# Associations Between Self and Peer's Fear of Fat in Predicting Dietary Restraint, Weight/Shape/Eating Concerns, and Thin‐Ideal Internalization: A Dyadic Exploration in Female Friend Pairs

**DOI:** 10.1002/eat.70160

**Published:** 2026-06-30

**Authors:** Shannon M. O'Connor, Caroline G. Martin, Leora L. Haller, Shannon Marhan

**Affiliations:** ^1^ Department of Psychology University of Toledo Toledo Ohio USA; ^2^ Department of Psychology Montclair State University Montclair New Jersey USA

## Abstract

**Objective:**

Fear of fat may reflect concern regarding rejection one may experience at a higher weight. Peers play a major role in socialization and support during adolescence and young adulthood. While studies have highlighted peers as an important source of influence underlying disordered eating, it is unclear whether one's own fear of fat or peers' fear of fat is associated with concerns about weight, shape, eating, and eating behaviors.

**Method:**

The present study used data from 94 female friend pairs (mean age = 20.18, SD = 1.77). Actor‐partner interdependence models were conducted to explore the extent to which one's own fear of fat predicted their own dietary restraint, weight concerns, shape concerns, eating concerns, and thin‐ideal internalization (actor effects), as well as the extent to which the individual's friend's fear of fat predicted the individual's dietary restraint, weight concerns, shape concerns, eating concerns, and thin‐ideal internalization (partner effects). The Antifat Attitudes Questionnaire was used to assess fear of fat. The Eating Disorder Examination Questionnaire was used to assess dietary restraint and weight/shape/eating concerns. The Sociocultural Attitudes Toward Appearance Questionnaire‐4 was used to assess thin‐ideal internalization. BMI was included as a covariate.

**Results:**

Findings demonstrated robust actor effects, whereas partner effects were small and/or non‐significant.

**Discussion:**

Results highlight that one's own attitudes about weight are stronger predictors of their dietary restraint, weight/shape/eating concerns, and thin‐ideal internalization. These results might further suggest that an individual's perception of their peers may be a stronger predictor of their disordered eating than their peers' objective attitudes.

## Summary

- An individual's fear of fat significantly predicted their own dietary restraint, weight concerns, shape concerns, eating concerns, and thin‐ideal internalization.
- Peer's fear of fat did not predict or marginally predicted the individual's disordered eating‐related constructs.
- Individual's own anti‐fat attitudes are stronger predictors of their eating and body image‐related concerns compared to their peer’s.

Fear of fat refers to an individual's anxiety over the idea of gaining weight or being overweight. This fear often stems from fear of stigmatization associated with overweight status (Dalley and Buunk 2009). Stereotypes that overweight and obese individuals are lazy, weak‐willed, unsuccessful, unintelligent, and lack self‐discipline are pervasive in Western culture (Puhl and Brownell 2001; Puhl and Heuer 2009). These stereotypes fuel real‐world consequences, as substantial research on weight bias illustrates that individuals with higher weights experience stigma and discrimination that may impact employment, income, education, media representation, romantic relationships, and both access to and treatment within healthcare settings (e.g., Fikkan and Rothblum 2012; Phelan et al. 2014; Sarlio‐Lahteenkorva et al. 2004). At its core, fear of fat may reflect a desire to avoid rejection from others (Moulton et al. 1998) and the negative consequences inherent in that rejection.

Late adolescence and young adulthood are developmental periods when, socially, peers play an influential role (Hartup and Stevens 1997; Laursen and Veenstra 2021). Whether due to socialization processes (i.e., exposure to peers leading to similarities; C. S. Crandall 1988) or selection processes (i.e., individuals selecting into like‐minded peer groups; O'Connor et al. 2016; Rayner et al. 2012), disordered eating appears to cluster within peer groups. Indeed, prior research has found similar levels of body image concerns, dietary restraint, the use of extreme weight‐loss behaviors (Paxton et al. 1999), and binge eating (Goldschmidt et al. 2014) within friendship groups. Few studies have explored the extent to which fear of fat may underlie these disordered eating cognitions and behavior and, further, whether an individual's fear of fat may influence their peers' body weight and shape concerns and dieting behavior.

Notably, prior research has suggested that interacting with a peer with explicitly anti‐fat attitudes can have detrimental effects. In an experimental study in which women with higher weights interacted with a same‐sex confederate with either explicit anti‐fat attitudes or unbiased attitudes, greater heart rate reactivity and anger were observed when interacting with a peer with anti‐fat attitudes. Further, perceived and objective high weight was associated with increased anticipated rejection, which was linked with poorer cognitive performance, lower state self‐esteem, and increased negative emotions, rumination, compensatory efforts, and thoughts related to anxiety and evaluation immediately after the encounter (Hunger et al. 2018). While Hunger et al. (2018) support the notion that interactions with a peer with explicit anti‐fat attitudes can trigger physiological and psychological stress reactions, this study used a confederate of similar age, gender, and race. It is unclear how naturally developed friends' fear of fat and anti‐fat attitudes more generally may impact their peers, and specifically, how it may impact one's thoughts about their own body weight, shape, eating, and body ideals.

To date, only one study has explored friends' fear of fat predicting restrained eating. As anticipated, one's own fear of fat was associated with one's own restrained eating; however, a friend's fear of fat did not predict one's restrained eating (Chow et al. 2019). Chow et al. (2019) focused on restrained eating; however, it is possible that peer groups with members who are concerned about weight gain (i.e., high “fear of fat”) may (1) have elevated weight, shape, and eating concerns and/or internalization of the “thin‐ideal” (i.e., the idea that the ideal beauty standard is of a thin figure with little body fat) and (2) may influence their peers' thoughts about their weight, shape, and eating, thin‐ideal internalization, and desired dietary behaviors.

The present study sought to explore this question by recruiting female friend pairs to participate together in a study on “exploring similarities and differences between friends on mood, personality, and behavior.” Prior research has often relied on a single informant to report on both their own and their peers' values around weight, shape, and eating. The recruitment of friend pairs allowed for more objective assessments of peers' values. The present study sought to explore the extent to which one's own fear of fat and one's friend's fear of fat predicted their weight concerns, shape concerns, eating concerns, thin‐ideal internalization, and dietary restraint. Notably, dietary restraint differs from restrained eating explored in past research (Chow et al. 2019), as dietary restraint taps a cognitive component associated with the intent to restrict one's eating regardless of the behavioral success of that intention. Thus, the present study expands past findings by exploring multiple cognitive aspects of disordered eating. Given past research demonstrates peers as an important source of influence on disordered eating (e.g., Eisenberg and Neumark‐Sztainer 2010; Keel et al. 2013; Meyer and Gast 2008), we hypothesized that underlying fear of fat would both predict one's own cognitions around weight, shape, eating, the thin‐ideal, and desire to restrict one's eating (i.e., dietary restraint), as well as their peer's disordered eating‐related outcome (i.e., weight concerns, shape concerns, eating concerns, thin‐ideal internalization, and dietary restraint).

## 1 Method

### 1.1 Participants

Our sample included 94 female–female friend pairs (mean age = 20.18, SD = 1.77) recruited via flyers administered via listservs, social media, posted around a university campus, and shared by professors unaffiliated with the project. Two individuals missing data indexing their fear of fat, 3 individuals missing BMI, and 15 individuals who completed the study without a partner or had a partner with missing data were excluded (*n* = 20 individuals total), resulting in a final analytic sample of 188 individuals, 94 complete dyads. Notably, no meaningful differences were found between individuals excluded due to missing data or a missing partner on our core study variables (Fear of fat: *t*(204) = −1.09, *p* = 0.28; Restraint: *t*(205) = 0.03, *p* = 0.98; Weight Concerns: *t(*205) = −2.09, *p* = 0.04; Shape Concerns: *t*(205) = −1.98, *p* = 0.05; Eating Concerns: *t*(205) = −1.17, *p* = 0.24; Thin‐Ideal Internalization: *t*(204) = −0.88, *p* = 0.38).

Majority of participants completed the study with their friend (98.9%) with the remaining pairs completing at different times (1.0%). Majority of participants completed the study in‐person in our lab space (71%). However, data collection occurred during the spring of 2022, when COVID‐19 was still a concern, and thus, the option to complete the study via Zoom was also provided, with 29% completing the study via Zoom. Friend pairs were separated either into separate physical rooms or separate Zoom rooms to complete informed consent and the study questionnaires independently. For individuals completing the study on Zoom, cameras were required to be turned on at the start of the assessment to confirm study participants appeared to match our study eligibility criteria: (1) female friend pairs, (2) aged 18–24 years, (3) English‐speaking, and (4) residing in the United States. Suspicious engagement in the study (e.g., refusing to turn on camera, unclear English‐fluency, etc.) was flagged, and sensitivity analyses were conducted to assess whether the removal of these individuals (*n* = 24 individuals; 12.8% of the sample) influenced study findings (see Table S1). Importantly, the majority of participants indicated that they were good friends (30.9%; “We are good friends. We frequently hang out and occasionally have deep conversations”) or best friends (62.2%: “We are best friends. We share everything with one another.”) with their participating friend. The remaining individuals classified their relationship as friends (5.9% “We are friends. We regularly hang out, but we don't know each other all that well.”) or acquaintances (1.1% “We are acquaintances. We know each other but are not close friends”). Duration of the friendship of the participating dyads was variable: 11.2% indicated less than 6 months, 13.8% 6 months–1 year, 12.8% 1–2 years, 13.8% 3–4 years, and 48.4% more than 4 years. The majority of participants indicated no familial relationship with their participating friend (92%), with 2.1% participating as siblings, 2.7% as cousins, and 3.2% as a different family relation. Further, 20.7% indicated their participating friend was their current roommate, 8.5% indicated they were past roommates, and 70.7% indicated never living together.

Approximately 30.9% of our sample identified as Hispanic/Latinx. Our sample was racially diverse, with 46.8% identifying as White, 26.6% as Black or African American, 8.0% as Asian, 1.6% as American Indian/Alaska Native, 1.1% as Native Hawaiian or Other Pacific Islander, 9.6% identified with a group not listed (most commonly writing in that they are Latinx or Middle Eastern), and 10.6% indicated they would not like to provide an answer. Notably, participants were allowed to select more than one race, and thus, percentages exceed 100%.

### 1.2 Measures

Fear of fat was assessed via a subscale on the *Antifat Attitudes Questionnaire* (C. S. Crandall 1994). The fear of fat subscale includes three items (e.g., “I worry about becoming fat”) scored using a Likert‐type response ranging from 0 (“very strongly disagree”) to 9 (“very strongly agree”). Item responses are summed, with higher scores indicating stronger anti‐fat attitudes. Excellent internal consistency has been demonstrated in past studies (α = 0.91; Chow et al. 2019), with good internal consistency found in the present sample (α = 0.88).

Dietary restraint, weight concerns, shape concerns, and eating concerns were assessed via the *Eating Disorder Examination Questionnaire (EDE‐Q 6.0*; Fairburn and Beglin 1994). The EDE‐Q assesses eating attitudes and behaviors over the past 28 days. All items used to calculate subscale scores are assessed on a 7‐point scale, with five items contributing to the restraint subscale (assessing attempts to refrain or avoid eating or follow dietary rules), five items contributing to the eating concerns subscale (assessing preoccupation with food, eating, and calories, fear of losing control over eating, concern around eating socially, and guilt about eating), eight items contributing to the shape concerns subscale (assessing dissatisfaction and preoccupation with the shape of one's body), and five items contributing to the weight concerns subscale (assessing dissatisfaction and preoccupation with one's weight). Each of these scales has demonstrated good to excellent internal consistency in past work (α = 0.81–0.91; Aardoom et al. 2012) and within the present sample (α = 0.82–0.90).

Thin‐ideal internalization was assessed with the *Sociocultural Attitudes Toward Appearance Questionnaire‐4* (SATAQ‐4; Schaefer et al. 2015). The SATAQ‐4 includes a 5‐item subscale that taps into thin/low body fat internalization. Items are assessed on a 5‐point Likert‐type scale and summed to create the subscale score. This subscale has demonstrated good internal consistency in females in the United States (α = 0.82–0.87; Schaefer et al. 2015) and in the present sample (α = 0.84).

Body mass index (BMI) was included as a covariate within analyses. BMI was calculated using participant self‐reported height and weight (BMI = weight [lb] × 703/height [in]^2^).

### 1.3 Data Analysis

Prior to analyses, the dyadic data were restructured, as friend pairs were considered indistinguishable dyads (i.e., the assignment of Friend A vs. Friend B was arbitrary). Thus, data were double‐entered into a pairwise data structure to allow for each friend's score to be listed in the Friend A and Friend B columns. Notably, restructuring the data allows for both Friend A and Friend B to have identical means and variances.

Actor‐partner interdependence models (APIM; Kenny et al. 2006; see Figure 1) were used to explore the extent to which an individual's own fear of fat predicted their disordered eating‐related outcome (actor effect) and the extent to which their friend's fear of fat predicted the individual's disordered eating‐related outcome (partner effect). APIMs used multi‐level modeling (with individuals [Level 1] nested within friend dyads [Level 2]) and generalized least squares with correlated errors and restricted maximum likelihood estimates (Kenny 2015). All predictors without zero as a possible outcome were grand‐mean centered (e.g., BMI). Separate APIM models were run for each disordered eating‐related outcome (i.e., restraint, eating concerns, shape concerns, weight concerns, and thin‐ideal internalization). To attempt to classify the type of interpersonal dynamic within the dyads, *k*‐values that index the ratio of partner effect to actor effect were calculated. Notably, *k*‐values exist on a continuum with *k* = 1 interpreted as a couple model indicating equal actor and partner effects, *k* = 0 as an actor‐only model (i.e., no partner effect), and *k* = −1 as a contrast model (i.e., actor and partner effects are similar in magnitude but opposite signs). In the analytic sample of *N* = 188, no individuals had missing data on fear of fat, any of the disordered eating‐related variables, or BMI.

**FIGURE 1:**
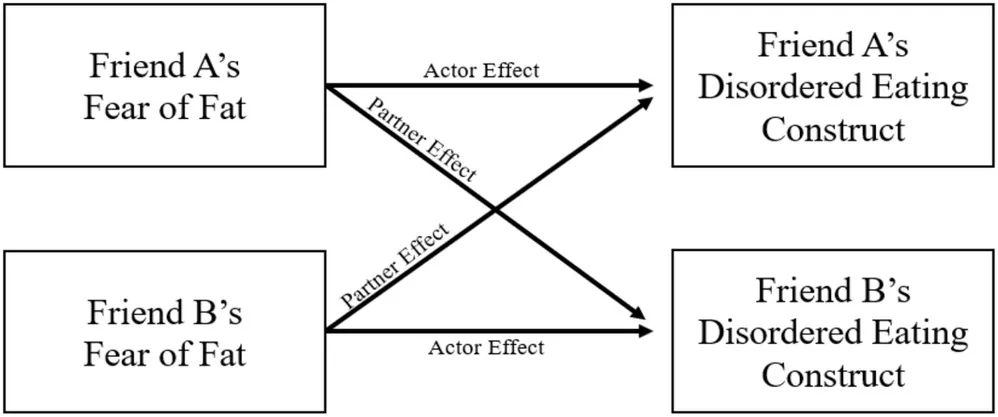
Sample actor‐partner interdependence model of friend's fear of fat and disordered eating constructs. This figure represents a sample actor‐partner interdependence model. Actor effects indicate associations between an individual's fear of fat (i.e., the actor) and their own disordered eating construct. Partner effects indicate associations between the friend's fear of fat (i.e., the partner) and the actor's disordered eating.

## 2 Results

### 2.1 Descriptive Analyses

Means, standard deviations, and bivariate correlations among predictor and outcome variables can be found in Table 1. Fear of fat and all the disordered eating‐related variables were normally distributed (all skewness and kurtosis values under 2). BMI was also normally distributed, with 6.9% of the sample indicating BMIs < 18.5, 43.1% with BMIs between 18.5 and 24.9, 25% with BMIs between 25.0 and 29.9, and 25% with BMIs of 30.0 or greater. Fear of fat demonstrated moderate‐to‐strong correlations with one's own outcome variables (*r*'s range from 0.44 to 0.59, all *p* < 0.001) and weak‐to‐moderate correlations with their friend's outcome variables (*r*'s range from 0.21 to 0.32, all *p* < 0.01). Notably, Pearson correlations do not control for non‐independence within the data and thus should be interpreted cautiously.

**TABLE 1:** Descriptive statistics and Pearson correlations (*N* = 188 individuals, 94 complete dyads).

	Mean	SD	1	2	3	4	5	6
1. Fear of fat (self)	9.90	7.02	—					
2. Restraint (self)	1.58	1.56	0.44***	—				
3. Weight concerns (self)	2.35	1.66	0.54***	0.60***	—			
4. Shape concerns (self)	2.61	1.65	0.59***	0.63***	0.92***	—		
5. Eating concerns (self)	1.27	1.33	0.47***	0.68***	0.78***	0.78***	—	
6. Thin ideal internalization (self)	10.35	5.24	0.49***	0.42***	0.57***	0.63***	0.49***	—
7. Fear of fat (friend)	9.90	7.02	0.30***	0.21**	0.32***	0.29***	0.24***	0.22**
8. Restraint (friend)	1.58	1.56	0.21**	0.16*	0.09	0.08	0.14	0.10
9. Weight concerns (friend)	2.35	1.66	0.32***	0.09	0.30***	0.23***	0.18*	0.24**
10. Shape concerns (friend)	2.61	1.65	0.29***	0.08	0.23***	0.17*	0.15*	0.18*
11. Eating concerns (friend)	1.27	1.33	0.24***	0.14	0.18*	0.15*	0.20**	0.15*
12. Thin ideal internalization (friend)	10.35	5.24	0.22**	0.10	0.24***	0.18*	0.15*	0.25***
13. BMI	26.25	6.69	0.21**	0.15*	0.39***	0.33***	0.26**	0.11

* *p* < 0.05.

** *p* < 0.01.

*** *p* < 0.001.

Intraclass correlation coefficients (ICC) were also calculated to assess the extent to which variance is attributable to dyad membership. ICCs range from 0 to 1, with scores closer to 1 reflecting strong agreement within dyads, whereas scores closer to 0 reflect that measurements within groups are as random as measurements from different groups. Overall, ICCs were small (dietary restraint: adjusted ICC = 0.06, weight concerns: adjusted ICC = 0.07, shape concerns: adjusted ICC = –0.10, eating concerns: adjusted ICC = 0.07, thin‐ideal internalization: adjusted ICC = 0.16), suggesting that 4%–16% of the variance across the various disordered eating outcomes is attributable to dyad‐level clustering.

### 2.2 Actor‐Partner Interdependence Models

#### 2.2.1 Dietary Restraint

Individual's fear of fat significantly predicted restraint (actor effect, *b* = 0.09, 95% CI: 0.06 to 0.12, SE = 0.02, *p* < 0.001, *ß* = 0.39); however, their friends' fear of fat did not significantly predict the individuals' restraint (partner effect, *b* = 0.02, 95% CI: −0.01 to 0.05, SE = 0.02, *p* = 0.20, *ß* =. 09). BMI did not significantly contribute to the model (*b* = 0.02, 95% CI: −0.02 to 0.05, SE = 0.02, *p* = 0.34, *ß* = 0.06). The *k* (i.e., ratio of partner/actor effects) was calculated as 0.22, which suggested results closer to an actor‐only model.

#### 2.2.2 Weight Concerns

Individual's fear of fat significantly predicted their own weight concerns (actor effect, *b* = 0.10, 95% CI: 0.07 to 0.13, SE = 0.01, *p* < 0.001, *ß* = 0.43); however, their friends' fear of fat also significantly predicted the individual's weight concerns (partner effect, *b* = 0.04, 95% CI: 0.01 to 0.07, SE = 0.01, *p* = 0.01, *ß* = 0.17). BMI was included as a covariate in the model and contributed significantly (*b* = 0.07, 95% CI: 0.04 to 0.10, SE = 0.01, *p* < 0.001, *ß* = 0.29). *k* was calculated at 0.40, which indicated this model falls in between an actor‐only model (partner effects = 0) and a couple model (*k* = 1).

#### 2.2.3 Shape Concerns

Individual's fear of fat predicted their own shape concerns (actor effect, *b* = 0.12, 95% CI: 0.09 to 0.15, SE = 0.01, *p* < 0.001, *ß* = 0.51). Friend's fear of fat also marginally predicted the individual's shape concern (partner effect, *b* = 0.03, 95% CI: 0.00 to 0.06, SE = 0.01, *p* = 0.04; *ß* = 0.12). BMI was included as a covariate in the model and contributed significantly (*b* = 0.05, 95% CI: 0.03 to 0.08, SE = 0.01, *p* < 0.001, *ß* = 0.21). *k* was calculated at 0.24, the model fits closer to an actor‐only model.

#### 2.2.4 Eating Concerns

Individual's fear of fat significantly predicted their own eating concerns (actor effect, *b* = 0.08, 95% CI: 0.05 to 0.10, SE = 0.01, *p* < 0.001, *ß* = 0.41). Friend's fear of fat did not significantly predict individual's eating concerns (partner, *b* = 0.02, 95% CI: −0.01 to 0.04, SE = 0.01, *p* = 0.12, *ß* = 0.10). BMI was included as a covariate in the model and contributed significantly (b = 0.03, 95% CI: 0.01 to 0.06, SE = 0.01, *p* = 0.01, *ß* = 0.17). *k* was calculated at 0.25, which suggested the model fits closer to the actor‐only model.

#### 2.2.5 Thin‐Ideal Internalization

Individual's fear of fat significantly predicted their own thin‐ideal internalization (*b* = 0.35, 95% CI: 0.25 to 0.44, SE = 0.05, *p* < 0.001, *ß* = 0.46). Friend's fear of fat did not significantly predict the individual's thin‐ideal internalization (*b* = 0.06, 95% CI: −0.04 to 0.15, SE = 0.05, *p* = 0.24, *ß* = 0.08). BMI did not contribute significantly to the model (*b* = 0.01, 95% CI: −0.09 to 0.12, SE = 0.05, *p* = 0.81, *ß* = 0.02). *k* was calculated at 0.17, which suggested the model fits closer to the actor‐only model.

## 3 Discussion

Findings from the present study highlight that one's own attitudes about weight are stronger predictors of their eating‐related thoughts and behaviors. As hypothesized, an individual's fear of fat significantly predicted their level of dietary restraint, weight concerns, shape concerns, eating concerns, and thin‐ideal internalization. Surprisingly, however, friends' fear of fat did not significantly predict elevated restraint, eating concerns, or thin‐ideal internalization for the individual. These findings suggest a lack of influence of friends' objective fear of fat on one's restraint, internalization of the thin ideal, and cognitions around eating.

Exceptions to this pattern were the findings for weight concerns and shape concerns. Indeed, small but statistically significant partner effects were demonstrated for weight and shape concerns. It is possible that a friend's fear of fat specifically elevates concerns around weight and shape for peers, relative to eating concerns or eating behaviors. It's also possible that there are temporal elements that are unable to be captured by the cross‐sectional design within the current study. More specifically, fear of fat may be associated with elevations in weight and shape concerns, which in turn later increase eating concerns, thin‐ideal internalization, and dietary restraint. It is also worth noting that the partner effects, while significant, were small and less than half the size of the actor effects. Thus, while small effects were shown for weight and shape concerns, the pattern of an individual's own fear of fat being more strongly predictive of their own weight/body‐related concerns is consistent across all disordered eating‐related constructs. Our findings were consistent with Chow et al. (2019), who found that women's fear of fat predicted their own but not their friends' restrained eating. However, our study extended these findings by demonstrating a similar pattern of effects for dietary restraint, weight concerns, shape concerns, eating concerns, and thin‐ideal internalization.

Past research that has explored the influence of a friend's value for topics around body weight, shape, and appearance has often relied on single informants to report on their peer's values and their own disordered eating‐related constructs. Within the present study, we have an objective assessment of a friend's true concern about weight gain. Integrating the present study's finding with past research may suggest that individuals' perception of their peers may be a stronger predictor of their disordered eating than their peers' objective attitudes, as we found non‐significant and/or small partner effects (i.e., peers' fear of fat largely did not significantly predict the individuals' eating‐related behavior or cognition). Notably, these findings do not necessarily invalidate past research using single informants, as it is likely that peers have some influence on one's eating and body‐related cognitions. However, future use of multiple informants may provide a more accurate view of the influence of peers and whether that influence is due to perception versus the actual values of one's peers.

Notably, the present study differed from prior studies that have explored the amount peer groups discuss subjects such as weight loss, body concerns, and appearance, as the present study only explored friends' fear of fat as a predictor. Fear of fat is an inward‐directed construct and thus does not necessarily reflect the messages that are outwardly communicated. Different results may be observed if we explored more externalized messages related to anti‐fat attitudes. Notably, Chow et al. (2019) found that individuals who reported less fear of fat reported less restrained eating unless they engaged in more negative body talk; however, the relationship between negative body talk and restrained eating did not differ based on friends' fear of fat (Chow et al. 2019). Taken together, even when modeling an externalization of fear of fat (e.g., negative body talk), a friend's fear of fat did not seem to influence the individual's restrained eating.

Interestingly, fear of fat predicts dietary restraint when explored in mother‐adolescent daughter dyads. Similar to the present study, Hart et al. (2021) found significant actor effects for both mothers and daughters (e.g., mothers' fear of fat predicted mothers' dietary restraint; daughters' fear of fat predicted daughters' dietary restraint). However, unlike the present study or Chow et al. (2019), partner effects were also significant. Mother's fear of fat significantly predicted daughters' dietary restraint. Daughter's fear of fat did not predict mother's dietary restraint; however, daughter's dislike of fat people did predict mother's dietary restraint (Hart et al. 2021). Notably, this study again focused on dietary restraint alone, and thus, it is unclear if similar results would be found for weight/shape/eating concerns or thin‐ideal internalization, but these findings suggest that another person's fear of fat can impact dietary behaviors. It is possible that the hierarchical dynamic with parent–child relationships, relative to more egalitarian dynamics within peer friendships, could help explain the differences across dyad types. Alternatively, the unique, long‐term, developmental relationship between mothers and daughters may explain differences in findings. Further research is needed to establish why these differences were observed.

Coupled with findings from past research, results from the present study may highlight that at least some peer influence may be driven by one's own perception of their peers, rather than an objective assessment of peers' values regarding body weight and shape. Network analysis findings demonstrate that fear of weight gain is central in psychopathology for bulimia nervosa (Levinson et al. 2017). Thus, interventions that focus on one's own fear of fat and the accuracy of one's perception of their friend's values may be beneficial relative to intervention at the peer group level. More specifically, individual interventions focused on increasing flexibility around perceived peer values may be helpful. Thought‐challenging and the introduction of cognitive distortions, such as mind‐reading of one's peers, may assist in increasing cognitive flexibility and decreasing the influence of one's perhaps inaccurate perception of one's peers. Further, future research could explore how one's own disordered eating may influence one's perception of their peers, given a core feature of clinical eating disorders is a distorted evaluation of the importance of weight/shape. This distorted view could influence one's perception of their peers' values.

While the present study has several strengths (e.g., data collected from multiple informants, exploration across several disordered eating‐related constructs), this study is not without limitations. First, while multiple informants were collected, questionnaires were still self‐report. Notably, there may be presentation biases in reports of anti‐fat attitudes, though the present study focused on an internally‐focused anti‐fat attitude (fear of fat), which may be more robust than an externalized anti‐fat attitude (e.g., “I have a hard time taking fat people too seriously”; C. S. Crandall 1994). Similarly, given a portion of our sample participated in our study virtually, self‐report height and weight were used to assess BMI. While past studies have demonstrated good agreement between measured and self‐reported weight and height, studies have also demonstrated some bias in self‐reported height and weight (see Fayyaz et al. 2024 for review). Second, the cross‐sectional nature of the data prevents causal inferences. It is plausible that weight/shape/eating concerns and thin‐ideal internalization may lead to fear of fat. Longitudinal approaches could help to clarify the directionality of these relationships. Third, given power limitations, we ran our APIMs separately in each disordered eating‐related outcome. Given the overlap between disordered eating‐related constructs (see Table 1), future research may benefit from modeling all disordered eating‐related outcomes simultaneously in one model to explore the extent to which actor's and partner's fear of fat predict the unique variance attributable to that specific disordered eating‐related outcome. Fourth, the present study focused on peer influence within female–female friend pairs. Results may not generalize to individuals in mixed gender dyads and/or dyads with individuals identifying as a gender other than female. Future research should explore peer influences in more gender‐diverse samples.

Moving forward, research using objective assessments of peers' views may help to clarify whether findings related to peers' influence completed with a single reporter are due to the individual's perception of their peers' values vs. their peers' true values. Findings in the present study suggest that one's own fear of fat is more impactful than a close friend's objective fear of fat. Therapeutic approaches may benefit from highlighting these findings, as individuals may benefit from challenging thoughts about their certainty of their friends' values regarding weight, shape, and appearance.

## Author Contributions

**Shannon M. O'Connor:** conceptualization, investigation, funding acquisition, writing – original draft, methodology, writing – review and editing, formal analysis, project administration, data curation, supervision. **Caroline G. Martin:** writing – review and editing, visualization. **Leora L. Haller:** conceptualization, writing – review and editing, methodology. **Shannon Marhan:** funding acquisition, writing – review and editing, conceptualization, project administration.

## Lived Experience Involvement Statement

No specific efforts were undertaken to involve persons with lived experience in the study design or execution, or in the preparation of this manuscript.

## Funding

Internal funding from Montclair State University awarded to Dr. O'Connor was used for participant compensation.

## Disclosure

Use of AI was not involved in any component of preparation of this manuscript.

## Conflicts of Interest

The authors declare no conflicts of interest.

## Supporting information


**Table S1:** Actor‐partner interdependent model sensitivity analyses (*N* = 164 individuals, 82 dyads).

## Data Availability

Data available on request from the authors.
