# Supplementary material for: Associations Between Self and Peer's Fear of Fat in Predicting Dietary Restraint, Weight/Shape/Eating Concerns, and Thin‐Ideal Internalization: A Dyadic Exploration in Female Friend Pairs
Source: Int J Eat Disord. 2026 Jun 30;59(10):2294–300. doi: 10.1002/eat.70160 (PMC13655968; doi:10.1002/eat.70160)
Supplement: Supplementary file 1 [file EAT-59-2294-s001.docx]

**Supplementary Table 1.** Actor-Partner Interdependent Model Sensitivity Analyses (N=164 individuals, 82 dyads)

| Model | Effect | *b* | *SE* | *β* | *p* |
| --- | --- | --- | --- | --- | --- |
| Restraint | **Actor** | **.10** | **.02** | **.46** | **<.001** |
|  | Partner | .02 | .01 | .06 | .44 |
|  | BMI | .02 | .02 | .07 | .37 |
| Weight Concerns | **Actor** | **.11** | **.01** | **.50** | **<.001** |
|  | **Partner** | **.03** | **.01** | **.14** | **.02** |
|  | **BMI** | **.07** | **.01** | **.30** | **<.001** |
| Shape Concerns | **Actor** | **.13** | **.01** | **.60** | **<.001** |
|  | Partner | .02 | .01 | .09 | .15 |
|  | BMI | **.05** | **.01** | **.20** | **<.001** |
| Eating Concerns | **Actor** | **.09** | **.01** | **.51** | **<.001** |
|  | Partner | .01 | .01 | .07 | .32 |
|  | **BMI** | **.03** | **.01** | **.18** | **.01** |
| Thin Ideal Internalization | **Actor** | **.41** | **.05** | **.55** | **<.001** |
|  | Partner | .04 | .05 | .05 | .44 |
|  | BMI | -.03 | .06 | -.04 | .60 |

*Note.* Predictor for all disordered eating outcomes is fear of fat. Dyads were treated as indistinguishable. Individuals flagged for suspicious engagement in the study were removed (n=24). Significant results are bolded.
